# Papillary Thyroid Cancer Trends in the Wake of the COVID-19 Pandemic: Is There a Shift toward a More Aggressive Entity?

**DOI:** 10.3390/diseases12030062

**Published:** 2024-03-20

**Authors:** Iyad Hassan, Lina Hassan, Farooq Bacha, Mohammad Al Salameh, Omran Gatee, Wiam Hassan

**Affiliations:** Department of Surgery, Burjeel Hospital, Abu Dhabi 7400, United Arab Emirates; thyroid@endocrine.ae (L.H.); farooq.bacha@burjeel.com (F.B.); mohamed.alsalama@hotmail.com (M.A.S.); omran.gatee@burjeel.com (O.G.); wiamh46@gmail.com (W.H.)

**Keywords:** PTC, thyroid cancer, COVID-19, pandemic, extrathyroidal extension, lymph vascular invasion, tall cell variant

## Abstract

Background: Globally, the incidence of papillary thyroid cancer (PTC) has been increasing over the last few decades and it has become the second most common cancer in women in the UAE. There is some evidence to suggest that COVID-19 infection might be directly linked to the development of aggressive variants of PTC. The primary goal of this study was to compare the clinical and pathologic characteristics of thyroid cancer patients treated at the largest endocrine surgery center in Abu Dhabi before and after the COVID-19 pandemic outbreak. Methods: This retrospective cohort analysis included patients who underwent elective thyroid surgery at Burjeel Hospital between January 2018 and December 2022. Patients were divided into two groups based on when the COVID-19 outbreak started: group one, comprising patients who had surgery between January 2018 and December 2019 (the “pre-pandemic group”), and group two, comprising patients who had surgery between January 2021 and December 2022 (the “post-pandemic group”). In addition to demographic data, clinicopathological factors, such as aggressive cell type, multifocality, tumor size and location, laterality, lympho-vascular invasion, and extrathyroidal extension, were assessed. We utilized the t-paired test for parametric variables and the Chi-square test for the cross-table analysis. Results: During the study, 1141 people had thyroid surgery, with an annual average of 285 procedures. PTC cases recorded in the final histopathological samples rose from 111 in the pre-pandemic era to 182 in the post-pandemic era. Neither the female-to-male gender ratio, which was 90:21 in the pre-pandemic group and 142:40 in the post-pandemic group (*p* = 0.532), nor the median age, which was 39.1 and 40.1 years, respectively, varied significantly between the two groups. However, there was a significant increase between pre-pandemic and post-pandemic in the aggressive PTC variants (3% vs. 11.5%, *p* = 0.001), increased poor prognostic factors such as bilateral multifocality (10.8% vs. 32.4%, *p* = 0.000), as well as increased capsule–vascular tumor invasion (19.8% vs. 27%); on the other hand, the size of the single foci was 17 mm in the pre-pandemic group compared to 13 mm in the post-pandemic group (*p* = 0.001). Conclusions: A significant rise in unfavorable prognostic markers and aggressive subtypes of PTC was seen post-pandemic in thyroidectomy patients operated on at a leading endocrine surgery center in the United Arab Emirates.

## 1. Background

Over the past five decades, there has been a significant rise in the incidence of thyroid cancer worldwide, with the United States experiencing a three-fold increase in diagnoses. While environmental and genetic etiologies have been proposed to explain these rising patterns, a growing body of research suggests that improved healthcare availability and the use of advanced diagnostic technologies are the main causes of increases in diagnosis [1]. On the other hand, it is believed that 70–80% of cases in the US and 50–90% in other affluent nations would not have caused symptoms, harm, or death if never identified [2]. Nevertheless, other authors have discussed a genuine rise in the occurrence of papillary thyroid cancer (PTC), together with a shift in its mortality rate. This indicates that overdiagnosis alone may not completely account for the observed patterns. The increasing incidence of thyroid cancer can potentially be explained by complex environmental, dietary, and/or genetic factors [3]. However, analyses of mortality rates specific to papillary thyroid cancer in the US have shown inconclusive findings. While one study indicated a consistent thyroid cancer mortality rate of approximately 0.5 deaths per 100,000 individuals between 1973 and 2002, another study using the same database revealed an annual rise in mortality of 1.1% overall and 2.9% specifically for papillary thyroid cancer between 1994 and 2013 [4].

There are several subtypes of PTC, some of which have a good prognosis. One of these is classic PTC, which accounts for 70–80% of cases and is defined by nuclear characteristics such as overlapping nuclei and nuclear clearance. Another subtype with an excellent prognosis is the follicular variant, which mimics follicular neoplasms but has nuclear characteristics. In contrast, the tall cell variant, with tall columnar cells and PTC nuclear features, and the diffuse sclerosing variant, with widespread infiltration, sclerosis, and lymph node metastasis, are the most common aggressive subtypes [5].

Treatment options for papillary thyroid cancer vary from active monitoring or local ablation procedures in low-risk instances to complete thyroidectomy with neck dissection, radioactive iodine therapy, and thyroid stimulating hormone suppression in aggressive and advanced cases. Nowadays, particular approaches are discussed in specialized endocrine tumor boards. The strategy may differ depending on the patient’s age, the size and spread of the tumor, and other health issues [6,7].

The rapid rise in patients confirmed to have COVID-19 compelled most healthcare systems worldwide to modify and prioritize hospital admissions starting in March 2020. Emergency measures and lockdowns, implemented between 15 March 2020 and 15 May 2020, constrained hospital entry for patients with chronic pathology, a situation observed globally and documented in various publications [8,9]. Specific protocols and guidelines were developed quickly, advising the postponement of various surgical treatments, including endocrine oncology [10,11,12]. Research on the direct impact of the COVID-19 pandemic on cancer aggressiveness is still ongoing, and findings are preliminary. However, several potential factors might contribute to changes in cancer progression and aggressiveness during the pandemic, such as delay in diagnosis and the disruption of treatment [13,14].

It is unclear whether the COVID-19 virus directly modifies the genome of thyroid cells or affects the immune response, both of which might lead to the development of more aggressive PTC. The fact that the virus penetrates human cells through the angiotensin-converting enzyme type 2 (ACE-2) receptor may help to answer this question. This receptor is expressed in many tissues, including the thyroid gland, and its expression suggests it may be vulnerable to infection by this virus [15].

The genomic composition of human cells may also be modified by the presence of COVID-19, potentially leading to more aggressive types of cancer such as thyroid cancer. In general, inflammation and oxidative stress induced by a COVID-19 infection can contribute to the development and progression of cancers, where they disrupt standard regulatory mechanisms controlling cell growth and differentiation [16,17]. For instance, viral infections may activate human oncogenes or interfere with tumor suppression systems, which is the most probable mechanism for COVID-19 to induce cancer [18]. COVID-19 infection may stimulate the production of nsp15. The cells responsible for producing nsp15 undergo hyperactivity and fast division [19]. Furthermore, the COVID-19 virus degrades the P53 tumor suppressor gene via its nsp3 protein. This results in decreased levels of P53, which is a predisposing factor for the development of cancer.

In the UAE, papillary thyroid cancer has become the second most common type of cancer in women over the past few decades. Abu Dhabi’s population enjoyed unhindered access to healthcare facilities throughout the COVID-19 pandemic, in stark contrast to the global situation [20]. The number of patients receiving surgical treatment at the Burjeel Endocrine Surgery Center remained consistent during this challenging period. The present study investigated the pathological characteristics observed in patients with papillary thyroid cancer who were operated on before and after the COVID-19 pandemic at the largest endocrine surgery center in the United Arab Emirates.

## 2. Materials and Methods

At a dedicated endocrine surgery center in the United Arab Emirates, a prospectively maintained surgical database for patients with thyroid disorders undergoing neck surgery was formed. Between January 2018 and August 2022, data were retrospectively evaluated from 1141 patients who underwent bilateral or unilateral thyroidectomy by a single endocrine surgeon (I.H.) at a tertiary institution. An average of 285 operations were conducted annually during the research period. A total of 293 patients whose final histopathologic results showed papillary thyroid cancer and who had at least 30 days of follow-up were included in our study. Patients with other types of thyroid malignancies were not included in the study. Standard operating room (OR) procedures for thyroid surgery at our institution, including patient placement on the OR table, selection of anesthesia, equipment setup, and intraoperative neuromonitoring, were previously described [21].

### 2.1. Surgical Technique

The surgical approach used in this study for minimally invasive open thyroidectomy included the use of general anesthesia without neuromuscular blocking, allowing for a comprehensive intraoperative neuromonitoring strategy. Following the patient’s transfer to the operating table, the following procedures were carried out: an ECG electrode, pulse oximeter, and blood pressure monitor were positioned, and baseline vital signs were recorded. After that, a cannula was inserted into the patient’s left hand. Subsequently, an intravenous (IV) dose of 2 mg of midazolam was given. For pre-oxygenation, an oxygenation mask was placed over the patient’s face, and the saturation was continuously controlled until it reached 100%. The patient was then given an injection of 20 mg of lidocaine, a bolus of propofol (dose calculated at 2.5 mg/kg), and a bolus of remifentanil (0.1 mg/kg). Ventilation was then administered using a Comfort Star^®^ anesthesia face mask from Dräger Medical AG in Lübeck, Germany. These masks were typically used for between one and three minutes. After that, an electromyogram endotracheal tube (EMG NIM^®^) was inserted and video laryngoscopy (C-MAC^®^, Storz, Tuttlingen, Germany) was used to ensure the EMG electrodes were placed correctly between both vocal cords. This was necessary for neuromonitoring throughout the operation. If the intubation process was problematic, it was possible to provide an additional propofol bolus, and the anesthesia could be maintained with the infusion of an inhalation anesthetic or opiate. Two centimeters above the sternal notch, a Kocher incision measuring 2.5–4 cm was cut along the skin fold. Before that, scissors were used to prepare the skin and platysma flaps on both the cranial and caudal sides in order to separate the strap muscles from the sternocleidomastoid above the clavicle. Electric equipment or energy would not be used at this time. Since all forms of the non-RLN (non-recurrent laryngeal nerve) branch from the vagal nerve above the point where the ITA and the vagal nerve crossed, medial retraction of the strap muscle and the thyroid lobe beneath it facilitated the visualization of the inferior thyroid artery (ITA) and middle thyroid vein. Subsequently, the vagal nerve (VN) inside the carotid sheath was seen, and neuromonitoring was used to rule out the potential of non-RLN (ADNM-VN1) below the inferior thyroid artery directly above the clavicle. A stimulus current of 1 mA was used; an EMG response greater than 200 µV was deemed adequate. When there was a negative signal, non-RLN was suspected; however, in order to rule out any technical issue or signal loss caused by anesthesia, the same process was carried out in the event of a positive signal, moving back to the right side to confirm the non-RLN. Next, bipolar cautery was used to remove the strap muscles from the thyroid. Additionally, ligaSureTM (Medtronic, Minneapolis, MN, USA) was used to divide the Kocher lateral veins and perform a lateral retraction of the strap muscles.

The thyroid lobe was gently removed from the paravertebral fascia, and the upper pole vessels were gently mobilized, very close to the capsule of the thyroid gland. When the superior laryngeal nerve (SLN) was tested with NIM, the results revealed a typical SLN curve smaller in amplitude than that of the RLN and VN. The thyroid gland was grasped with a Kocher clamp very near the upper pole vessels and raised from the prevertebral facia distally and laterally in order to see and protect the SLN. At this stage, in order to rule out any neural structures from the intended ligation, the upper pole vessels were divided into two or three short lengths and ligated using LigaSureTM after previous testing with NIM. The SLN and/or non-RLN were immediately cooled with 5 mL of cold saline to stop the heat damage from spreading. The whole vagal nerve, from the level of the larynx’s incisura to the clavicle bone, could then be tested since the right lobe could be luxated into the small incision. A positive signal could then be observable at the larynx incisura level using non-RLN. To locate and dissect the non-RLN, the thyroid and larynx were retracted medially, and the carotid sheath was retracted laterally. The use of NIM assisted in this process. A non-RLN was traced from its origin on the vagus nerve until it entered the cricothyroid membrane. Typically, during this phase, the upper parathyroid gland would be discovered and protected. A signal was acquired by locating the laryngeal recurrent nerve below the inferior thyroid artery after mobilizing the whole lobe in the wound using typical anatomical techniques. The NIM signal located below ITA was officially recorded as ADNM-RLN-1. The lower pole vessel was identified and then separated using LigaSure™. Maintaining vascular supply also helped to preserve the lower parathyroid.

### 2.2. Data Acquisition

The prospective surgical database combined preoperative, intraoperative, and postoperative clinicopathological criteria that were particularly relevant to the progression of thyroid cancer. Standard patient characteristics, laboratory findings, and imaging investigations were also included in this resource. In addition to the typical non-cancer-specific factors, such as results of calcium and thyroid function tests, tumor markers, multifocality, laterality, size of the tumor in millimeters, subtypes of PTC, and capsular or vascular invasion were the benchmarks used to evaluate the aggressiveness of the tumor. Upon completing the microscopic review, the results were evaluated and double-checked by at least one additional qualified pathologist at our institution. They described the types of papillary thyroid cancer as the classic or traditional papillary thyroid carcinoma or the tall cell variant. The classic papillary thyroid carcinoma is well known among clinicians and pathologists as the type with the best prognosis. Its diagnosis criteria are as follows:

Papillary architecture: Tumor cells protrude into thyroid follicles in a papillary growth pattern. The formations are called “papillae”.

Nuclear features: PTC cells display nuclear expansion, clearing (ground-glass appearance), grooves, and pseudoinclusions. The “Orphan Annie eye” nuclei of PTC cells are widely mentioned.

Psammoma bodies: Tumor stroma calcifications are concentric and lamellated. Classic PTC has psammoma bodies.

The most common aggressive variant of papillary thyroid cancer is the tall cell variant histopathology, which is characterized by tall and columnar-shaped cells with a height-to-width ratio greater than 3:1 and clinical features associated with a higher risk of extrathyroidal extension, lymph node metastasis, and recurrence compared to classic PTC. Aggressive forms of papillary thyroid carcinoma are linked to unfavorable prognostic markers, including metastatic and recurrent disease, as well as overall survival.

### 2.3. Study Groups

In contrast to other research works that have investigated the possible effects of the pandemic on thyroid cancer, we divided patients into two distinct groups. The group of 111 patients who underwent surgery for thyroid-related conditions in the two years before the period of lockdown, mainly in 2018 and 2019, was referred to as the pre-pandemic group. The post-pandemic group comprised 182 patients who had operations in the years 2021 and 2022. This decision about how to divide patient groups was determined because vaccinations began in January of 2021. The year 2020, designated as the year of the pandemic, was documented; nevertheless, it was excluded from the direct comparison due to its abbreviated duration (commencing on 15 March). Additionally, the total number of COVID-19-infected individuals in the UAE during that year amounted to only 500 cases. Moreover, our hypotheses posited that the direct influence of the virus in inducing aggressive cancer required a longer duration to manifest.

### 2.4. Statistical Analysis

Student’s *t*-test was used to compare the means of continuous variables. When the sample size was small, we used the Chi-squared test to compare continuous variables and Fisher’s exact test for categorical variables. Non-parametric variables were compared between groups using the Mann–Whitney U test. ANOVA was used to test for differences among the means of the two patient groups. SPSS 29.0 was used for all statistical testing (IBM, SPSS^®^, Chicago, IL, USA). To draw conclusions from the data, a *p* value of less than 0.05 was considered statistically significant.

## 3. Results

**Figure 1:**
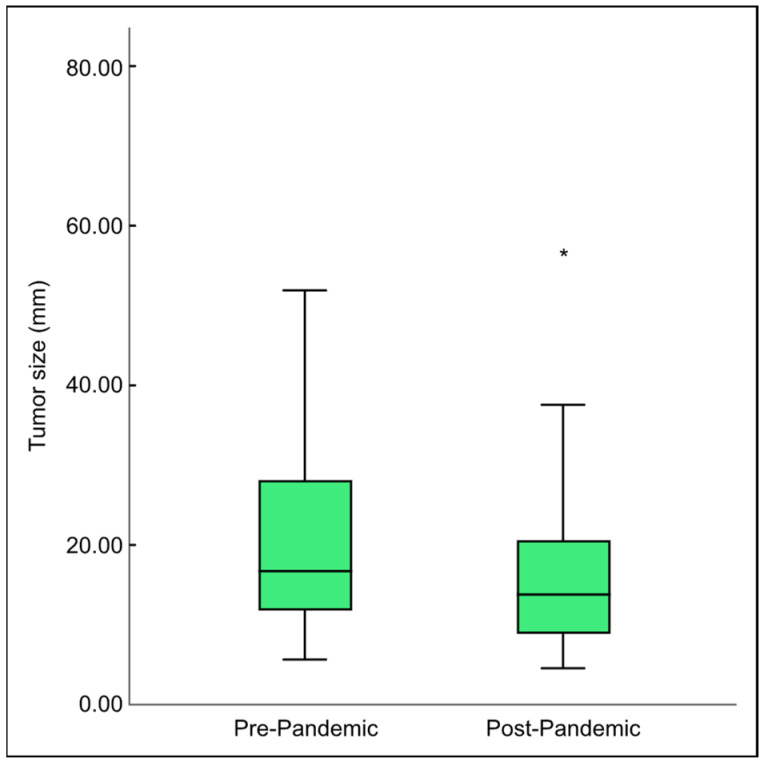
Comparison of the median tumor size found in final histopathology before and after the COVID-19 pandemic. * indicates significance using the *t*-test with *p* = 0.001.

**Figure 2:**
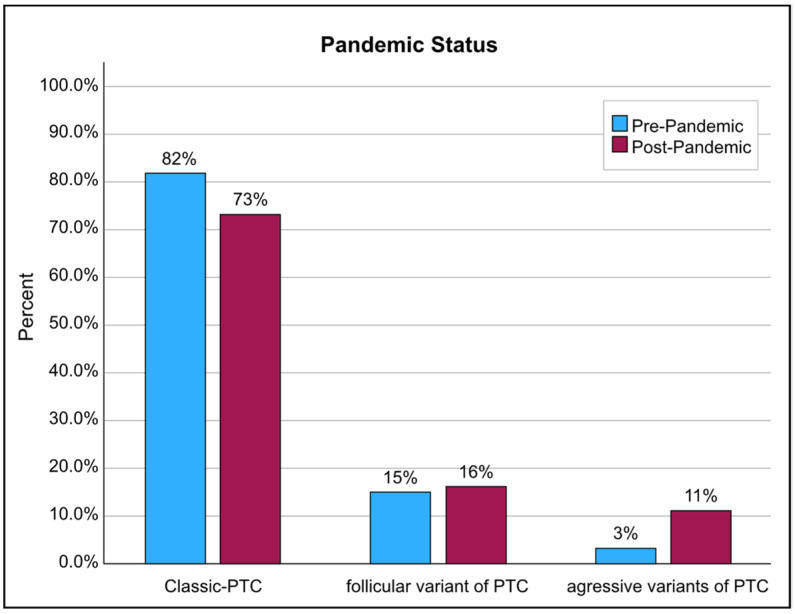
The rate of aggressive, poor prognosis tall cell PTC in the final histopathology, before and after the COVID-19 pandemic, in comparison with classic and the better-prognosis follicular forms.

Between 2018 and 2021, an average of 56–55 cases of thyroid cancer were detected in histopathological specimens annually. By 2022, however, this figure jumped to 132 cases. The ratio of females to males was 90:21 in the pre-pandemic group and 142:40 in the post-pandemic group (Mann–Whitney U-test, *p* = 0.532), and the median age was 39.1 and 40 years, which did not differ significantly between the two groups (*t*-test, *p* = 0.511). The histopathology features showed a significant decrease in the size of the largest tumor foci in the post-pandemic era (Figure 1). However, aggressive variants of PTC, mainly the tall cell variant, increased (3% pre-pandemic vs. 11.3% post-pandemic; Chi-square test, *p* = 0.017) (Figure 2). Furthermore, the poor prognostic factors, including bilateral multifocality (10.8% pre-pandemic vs. 32.4% post-pandemic; Chi-square test, *p* = 0.001) and capsule–vascular invasion (19.8% pre-pandemic vs. 27% post-pandemic; Chi-square test, *p* = 0.002), also escalated (Table 1).

## 4. Discussion

The COVID-19 pandemic had a profound impact on the care provided to cancer patients in several nations. Some cancer patients were told to limit their hospital visits to prevent nosocomial COVID-19 infections. Thus, many cancer patients may have delayed their treatment because of COVID-19. These patients may have experienced severe levels of anxiety and worry. Some studies and recommendations have suggested that waiting three months from diagnosis for thyroid cancer treatment is acceptable [19,20,21,22]. However, this suggestion should be treated with caution. Tissue-specific malignancies require individualized treatment plans due to their varying malignancy and aggressiveness. If treatment for diseases such as melanoma, non-small-cell lung cancer, or acute myeloid leukemia is delayed, the patient’s prognosis worsens significantly [10,23]. In contrast, patients with indolent tumors, such as most prostate cancers or differentiated thyroid carcinomas, may be able to wait for a while before experiencing any signs of malignancy [21,24]. Current clinical research has primarily addressed the dismal prognosis for high-risk tumors, but data for low-risk tumors are scarce. The American Thyroid Association proposed active surveillance to determine the growth rate of papillary thyroid cancers, opposing surgery as the immediate intervention since there is a research gap in how these malignant cells grow and develop [2,25,26].

The incidence of PTC is estimated to be between 80% and 95%, making it the most common form of thyroid cancer. Its incidence is also rising alarmingly quickly. There is an ongoing debate about whether or not the rapid increase in PTC prevalence may be attributed to overdiagnosis driven by recent advances in medical technology and improved access to healthcare facilities or whether there is a true rise in cancer incidence due to external factors [27,28,29]. However, 20% and 10% of differentiated thyroid cancer patients are at risk of local recurrence and distant metastases, respectively, and two-thirds lose RAI uptake, in what is termed radioactive iodine-refractory-differentiated thyroid cancer. Due to the restricted range of available therapeutic choices, the prognosis for these patients is poor, with a 10-year survival rate of less than 10% [30,31,32].

When the post-pandemic group was compared to the pre-pandemic group, the current study found that the number of thyroid surgeries had increased and that the cancer rate in the final histopathology was not only higher, but also more invasive and had more aggressive pathological findings. This finding partially aligned with findings from Asia and Romania, which described a reduction in total thyroidectomy numbers but an increase in aggressive thyroid variants [8,23,26]. However, the rise in cancers with more aggressive behavior cannot be explained by delayed diagnosis and treatments for two reasons: firstly, the circumstances of COVID-19 management in Abu Dhabi, where there was no restriction for citizens to access healthcare facilities; secondly, the fact that PTC is relatively slow-growing and may be survived without immediate treatment, as reported by Ito et al. in the biggest prospective series of active surveillance patients at Kuma Hospital over 22 years. Ito et al. found that 340 of 1395 trial patients chose active surveillance over immediate surgery, with identical oncologic results, and only 8% of active surveillance patients had 3 mm or greater tumor growth over 10 years. Over the same timeframe, 3.8% of patients developed new lymph node metastases [1].

Clearly, the clinical findings might be interpreted in various ways. One might simply think that thyroid cancers have become more aggressive over time. While the current study found that tumor sizes decreased, they grew more aggressively and bilaterally multifocal. We could not exclude the working premise that the incidence increased synchronously with the COVID-19 pandemic. Patient anxiety should also be taken into account. According to earlier research, patients with more aggressive forms of thyroid cancer have been shown to worry more [33,34]. After the lockdown, anxious patients may have sought medical attention more frequently, exaggerating the perceived severity of the disease. Furthermore, although thyroid tumors are typically slow-growing, some patients may have experienced tumor development due to the lockdown imposed by the epidemic [15].

Another proposed theory is that survivors of severe COVID-19 may be at risk for developing cancer because the cytokine storm stimulates important signaling pathways linked to abnormal cell proliferation and may hamper the immune system’s response to malignancies. Most people who catch COVID-19 only experience mild symptoms, although this highly contagious cytokine storm can be triggered by COVID-19 infection, which further facilitates viral entry and hastens immune evasion [35]. However, recent research has shown that infection with COVID-19 needs the receptor angiotensin-converting enzyme 2 and transmembrane protease serine 2 to recognize the S protein [36]. Angiotensin-converting enzyme 2 and transmembrane protease serine 2 are significantly expressed in the thyroid, making it vulnerable to virus attacks. Since ribonucleic acid (RNA) from COVID-19 was found in the thyroid, this finding strongly suggests that COVID-19 can infect the thyroid and might be able to alter its genomic material [37,38,39,40,41]. Nevertheless, thyroid cancer following COVID-19 illness has received increasing research interest, since the thyroid is an endocrine organ that the immune system can damage. Whether the occurrence rate rises after persistent COVID-19 inflammatory conditions, a known risk factor for cancer, is also uncertain.

Parallel concepts about other viruses and human malignancies are relevant here. Although the human papillomavirus, hepatitis B and C viruses, and the Epstein–Barr virus (EBV) are all known to cause human malignancies, thyroid cancer is not commonly considered to be a classical type of cancer that is connected with viruses [42]. However, the question of whether or not COVID-19 can cause an increase in the incidence of thyroid cancer is speculative. Despite this, the current study showed not only an increase in thyroid cancer cases operated on in our center, but also a rise in the aggressive parameters found in final pathologies, such as bilateral multifocality, tall cell variants, and capsular and vascular invasion. These interesting findings occurred just one year after the COVID-19 outbreak. The pandemic and its related factors, such as psychological stress, infection, and transmission, are the only variables that could be responsible for this new finding.

Our research had some limitations. The primary limitation was the absence of data on patients who tested positive for COVID-19 and had a clinically significant COVID-19 infection before thyroidectomy. The primary obstacle to documenting this crucial metric was mostly a cultural one. Our clinical records lacked certain variables, such as a patient’s history of COVID-19 infection, the extent to which that infection had progressed, whether or not it had returned, and whether or not a vaccine had been administered. In addition, patient numbers in the comparisons between the pre-pandemic and post-pandemic periods were insufficient for more conclusive results. Due to this being a retrospective study conducted at a single location, the applicability of our theory is restricted. Additional prospective studies, ideally with larger sample sizes from different regions and focusing on molecular and biological research, are required to evaluate our findings.

## 5. Conclusions

This study compared individuals diagnosed with thyroid cancer who were treated at a high-volume endocrine surgical center in Abu Dhabi, the United Arab Emirates, before and after the COVID-19 pandemic. Our findings suggested that the pandemic significantly affected thyroid cancer pathological features by increasing poor prognostic factors, such as aggressive types, bilateral multifocality, and capsule–vascular invasion. This could not be explained by delays in diagnosis and treatment since access to healthcare facilities in Abu Dhabi was never restricted in any way during the pandemic. This finding contradicted widespread assertions proposed by recent studies that attributed this new observation to lockdowns, delayed diagnosis, and delayed treatment. A multifactorial cause for the increase in cases as well as pathological behavior needs further evaluation to determine if the SARS-CoV-19 virus has a direct or indirect cancerogenic effect on the thyroid gland, together with other factors such as psychological stress. However, given the inherent limitations of this retrospective monocentric study, it is prudent to treat with caution the conclusions drawn from our findings. The results, though intriguing, should be viewed as preliminary observations, and their biological significance warrants further exploration in dedicated studies. We acknowledge the need for caution in generalizing these findings and emphasize the importance of future research endeavors to validate and expand upon our observations.

## Figures and Tables

**Table 1:** Comparison of clinical and pathological features before and after the pandemic. **▪** and † denote a statistically significant difference between the groups, as determined using a Chi-square test. * denotes a statistically significant difference between the groups, as determined using a *t*-test. (*n*) is the absolute number of cases.

	Pre-Pandemic	Post-Pandemic	Total	*p*-Value
Gender, female/male (*n*)	90/21	142/40	232/61	0.532
Age in years	39.1	40.1		0.669
Median tumor size in mm	18.1 ▪	13.1		0.001
Multifocality unilateral/bilateral (*n*)	99/12	123/59 ^†^	222/71	0.001
Noninvasive (*n*)	89	133	222	0.232
Capsule–vascular invasion (*n*)	22	49 ^†^	71	0.001
Lobectomy/total thyroidectomy	20/91	39/143 *	59/234	0.001

## Data Availability

The data that support the results of this study can be requested from the corresponding author.
